# Selection of Reliable Reference Genes for Gene Expression Studies of a Promising Oilseed Crop, *Plukenetia volubilis*, by Real-Time Quantitative PCR

**DOI:** 10.3390/ijms160612513

**Published:** 2015-06-03

**Authors:** Longjian Niu, Yan-Bin Tao, Mao-Sheng Chen, Qiantang Fu, Chaoqiong Li, Yuling Dong, Xiulan Wang, Huiying He, Zeng-Fu Xu

**Affiliations:** 1 School of Life Sciences, University of Science and Technology of China, Hefei 230027, China; E-Mail: niulongjian@126.com; 2 Key Laboratory of Tropical Plant Resources and Sustainable Use, Xishuangbanna Tropical Botanical Garden, Chinese Academy of Sciences, Menglun 666303, China; E-Mails: taoyanbin@xtbg.ac.cn (Y.-B.T.); chenms@xtbg.org.cn (M.-S.C.); qtfu2002@163.com (Q.F.); yuling-dong@163.com (Y.D.); wangxiulan@xtbg.ac.cn (X.W.); hhy@xtbg.org.cn (H.H.); 3 Department of Life Sciences, College of Life Science and Agriculture, Zhoukou Normal University, Zhoukou 466001, China; E-Mail: lcq2013@aliyun.com

**Keywords:** *Plukenetia volubilis*, reference gene, RT-qPCR, flower development, seed development, biofuels

## Abstract

Real-time quantitative PCR (RT-qPCR) is a reliable and widely used method for gene expression analysis. The accuracy of the determination of a target gene expression level by RT-qPCR demands the use of appropriate reference genes to normalize the mRNA levels among different samples. However, suitable reference genes for RT-qPCR have not been identified in Sacha inchi (*Plukenetia volubilis*), a promising oilseed crop known for its polyunsaturated fatty acid (PUFA)-rich seeds. In this study, using RT-qPCR, twelve candidate reference genes were examined in seedlings and adult plants, during flower and seed development and for the entire growth cycle of Sacha inchi. Four statistical algorithms (delta cycle threshold (Δ*C*_t_), BestKeeper, geNorm, and NormFinder) were used to assess the expression stabilities of the candidate genes. The results showed that *ubiquitin-conjugating enzyme* (*UCE*), *actin* (*ACT*) and *phospholipase A22* (*PLA*) were the most stable genes in Sacha inchi seedlings. For roots, stems, leaves, flowers, and seeds from adult plants, *30S ribosomal protein S13* (*RPS13*), *cyclophilin* (*CYC*) and *elongation factor-1alpha* (*EF1α*) were recommended as reference genes for RT-qPCR. During the development of reproductive organs, *PLA*, *ACT* and *UCE* were the optimal reference genes for flower development, whereas *UCE*, *RPS13* and *RNA polymerase II subunit* (*RPII*) were optimal for seed development. Considering the entire growth cycle of Sacha inchi, *UCE*, *ACT* and *EF1α* were sufficient for the purpose of normalization. Our results provide useful guidelines for the selection of reliable reference genes for the normalization of RT-qPCR data for seedlings and adult plants, for reproductive organs, and for the entire growth cycle of Sacha inchi.

## 1. Introduction

Sacha inchi (*Plukenetia volubilis* L.), a member of the Euphorbiaceae, is native to the rain forest of South America [1,2]. Because its seed oil is rich in polyunsaturated fatty acids (PUFAs) and lipovitamins, Sacha inchi has great potential economic value to the food and pharmaceutical industries [3,4]. Moreover, Sacha inchi oil is also a good feedstock for biodiesel production [5]. To promote gene function studies in Sacha inchi, transcriptomic analysis has been performed for the period of seed development, and numerous key genes involved in the regulation of seed oil biosynthesis have been identified [6]. A good characterization of expression profiles of these key genes will facilitate a better understanding of gene function in seed oil biosynthesis.

Characterized by high sensitivity, specificity and accuracy, real-time quantitative PCR (RT-qPCR) has become the preferred method for detecting and measuring gene expression [7,8,9]. A prerequisite for the reliable analysis of gene expression is the normalization of RT-qPCR data, which can minimize the non-specific variations caused by variations in the quantity and quality of mRNA and variations in the efficiencies of reverse transcription and PCR [10,11,12,13]. Therefore, the selection of appropriate reference genes as internal controls that are expressed at constant levels among tissues and over time is very important.

In the last decade, several statistical algorithms have been developed for the selection of reference genes for RT-qPCR analysis, such as the delta cycle threshold (Δ*C*_t_) [14], geNorm [15], BestKeeper [16] or NormFinder [17] algorithms. The Δ*C*_t_ method ranks the candidate genes by comparing the relative expression of pairwise under a given set of experimental conditions [14]. The Δ*C*_t_ method indicated the mean of standard deviation (SD) of each candidate reference genes, and the candidate with the lowest SD value was proposed to be the most stable gene [14]. The geNorm is a Visual Basic application tool that relies on the principle that the expression ratio of two perfect reference genes should be constant under different development stages or in various plant tissues. The expression stability (M) is calculated based on the average pairwise variation between all reference genes tested. The gene with a lower M value indicated the gene expression is more stable [15]. The BestKeeper program evaluates the most stably expressed genes based on the coefficient of variance (CV) and SD of the quantification cycle (Cq) values. The lower coefficient of variance and standard deviation (CV ± SD) indicated the gene expression was more stable [16]. The NormFinder program is based on a variance estimation approach, which ranks the candidate genes according to the stability of a gene under a given set of experimental conditions compared to the rest of the tested genes. The more stably expressed genes are indicated by the lower average expression stability values (M values) [17]. The application of these algorithms has simplified the identification of reliable reference genes by enabling the rapid calculation of the expression stability and the determination of the optimal number of reference genes required for normalization [18,19].

The identification of optimal reference genes for RT-qPCR has been reported for several plants, including bamboo [20], *Jatropha curcas* [21], coffee [22], oil palm [23], peach [24] and *Petunia hybrida* [25]. However, a number of commonly used housekeeping genes, such as *actin* (*ACT*), *elongation factor 1alpha* (*EF1α*), *glyceraldehyde-3-phosphate dehydrogenase* (*GAPDH*), and *ubiquitin*(*UBQ*), is insufficient for RT-qPCR normalization because of variations in expression in different species, tissues, developmental stages or environmental conditions. For sesame, *SiACT* was recommended as the reference gene for seed development and germination, although *SiUBQ6* was better for bud development [26]. For Chinese cabbage, *EF1α* was reported to be the best reference gene among five tissues, and *GAPDH* was most suitable for drought stress conditions [27]. The *18S rRNA* (*18S*), *ACT* and *GAPDH* genes were reported to be expressed unstably in papaya (*Carica papaya*) under numerous experimental conditions [28]. Hence, the selection of multiple housekeeping reference genes is required for the accurate normalization of gene expression levels under varied experimental conditions.

For this study, in order to reduce the likelihood that the reference genes exhibited regulated co-variation, a group of genes with varied roles in different cellular processes were chosen (Table 1). The expression stabilities of twelve candidate reference genes (*18S*, *ACT*, *CYC*, *EF1α*, *GAPDH*, *PLA*, *RPII*, *RPS13*, *TEF2*, *TUB*, *UBL* and *UCE*) were examined in Sacha inchi seedlings and adult plants, during flower and seed development, and for the entire growth cycle of Sacha inchi. Our results indicate that traditional housekeeping genes were less stably expressed than other reference genes in the given experimental datasets.

**Table 1:** Selected candidate reference genes, primer sequences and PCR amplification characteristics.

Gene/GenBank Accession Number	Description	Function	Forward (F) and Reverse (R) Primer Sequences (5′→3′)	Amplicon Length	*T*m (°C)	Amplification Efficiency (%)	Correlation Coefficient
*18S*/KP729648	18S ribosomal RNA	ribosomal structure	F: ACCAGGTCCAGACATAGTAAGGATTGA	140 bp	81.73	106.40	0.999
			R: AGTTAGCAGGCTGAGGTCTCGTT				
*ACT*/GADC01011038	actin	cytoskeletal structural protein	F: CCAGAAGTCTTGTTCCAGCCATCTC	185 bp	80.66	105.78	0.999
			R: GCGGTGATCTCCTTGCTCATACG				
*CYC*/GADC01018836	cyclophilin	protein folding	F: GGCAAGATACGAACGGATCACAGTT	145 bp	82.95	108.93	0.999
			R: GGCACTCCACTCCGACTTCCTT				
*EF1α*/GADC01006492	elongation factor 1-alpha	protein biosynthesis	F: GGTATTCTCAAGCCTGGTATGGTTGT	102 bp	80.48	94.98	0.999
			R: GAGAGCCTCCTGAAGAGCCTCAT				
*GAPDH*/GADC01052274	glyceraldehyde-3-phosphate dehydrogenase	glucose metabolism	F: TGGCAAGCATATTCAGGCAGGAG	116 bp	81.63	94.98	0.999
			R: TTGGCTCATCAGGATTGTAGGTATCAG				
*PLA*/KP729647	phospholipase A22	lipid catabolic process	F: ATACCATACAGAACGCAGCTTGTGAA	101 bp	79.92	103.33	0.998
			R: TTCCGCCAGTTCCAACCTATCCA				
*RPII*/GADC01020629	RNA polymerase II subunit	mRNA process	F: GCCTCGGTCTCATTCCTCTTACAAG	109 bp	82.44	104.17	0.999
			R: AACTCAACAGAACAATACTCGCACTGA				
*RPS13*/GADC01008223	30S ribosomal protein S13	DNA-templated transcription	F: TAATGCACAGCTTCCAGATGAC	202 bp	81.47	90.55	0.999
			R: AACCAGTCGCTTTGATTCTTCT				
*TEF2*/GADC01000224	transcription elongation factors-II	transcription	F: AGATTCAGAGCATGAAGAGGGAC	182 bp	82.18	104.17	0.996
			R: CGATCGGTATTTGTTGCGATTT				
*TUB*/GADC01018931	Tubulin beta-4 chain	structural constituent of cytoskeleton	F: ACAATTCACTGCCATGTTCAGGAGAA	169 bp	82.05	97.83	0.999
			R: GTCATCTTCGTAGTCACCTTCGTCATC				
*UBL*/GADC01024109	ubiquitin-like	protein binding	F: GCTACGTCTGCGTGGAGGAATG	197 bp	82.39	99.53	0.996
			R: TGTAGTCTGCCAATGTGCGTCC				
*UCE*/GADC01034781	ubiquitin-conjugating enzyme	ubiquitin-dependent protein catabolic process	F: TGGAATGGATGACGGAGACGACAT	142 bp	78.74	100	0.997
			R: AACACTTGGTGGCTTCTCTGGATAATC				

## 2. Results

### 2.1. Specificity and Efficiency of PCR Amplification of the Candidate Reference Genes

**Figure 1:**
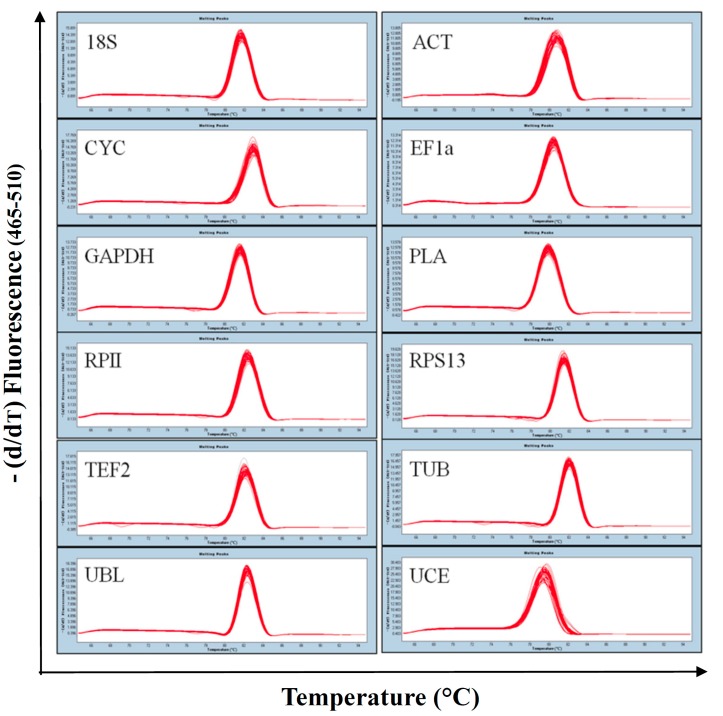
**Figure** **1.** Melting curves for the twelve candidate reference genes. The melting temperature of each amplicon is visualized by plotting the negative derivative of the change in fluorescence divided by the change in temperature in relation to the temperature (−(d/dT) Fluorescence (465–510)).

A total of twelve candidate reference genes (*18S*, *ACT*, *CYC*, *EF1α*, *GAPDH*, *PLA*, *RPII*, *RPS13*, *TEF2*, *TUB*, *UBL* and *UCE*) were selected to normalize the gene expression levels in Sacha inchi using RT-qPCR. The specificity of the primers (Table 1, Supplementary Figure S1) was confirmed by the single peak melting curves of the qPCR products (Figure 1) and the presence of a single band at the correct size for each primer pair in 2% agarose gel electrophoresis (Supplementary Figure S2). The melting temperatures of the PCR products all ranged between 78.74 °C for *UCE* and 82.95 °C for *CYC* (Table 1). The amplification efficiencies ranged from 90.55% for RPS13 to 108.93% for *CYC*, and the correlation coefficients (*R*^2^) for the primers all ranged between 0.996 and 0.999 (Table 1).

### 2.2. Transcript Accumulation of Candidate Reference Genes

**Figure 2:**
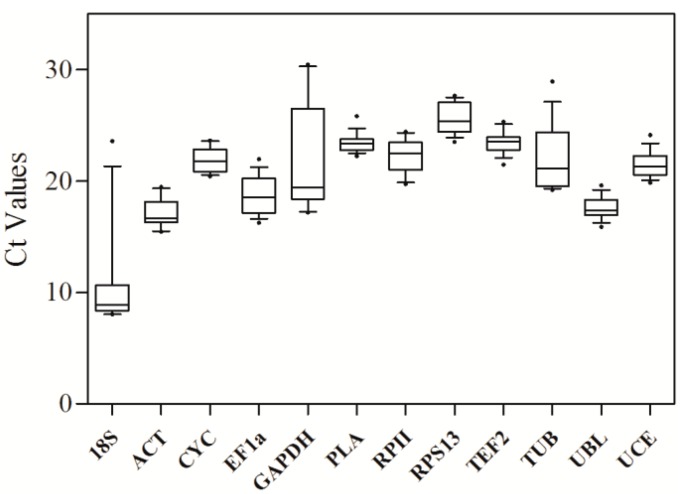
Average cycle threshold (*C*_t_) values for the twelve candidate reference genes. Boxes indicate the interquartile range. Lines across the boxes indicate the average *C*_t_ values. Whiskers represent 95% confidence intervals, and black dots represent outliers.

The transcript levels of the twelve candidate reference genes, presented as the cycle threshold (*C*_t_) values, were obtained by RT-qPCR. The box-plot analysis was performed using GraphPad Prism 5 software (GraphPad Software, San Diego, CA, USA). The data used to produce the box-plot were shown in the supplementary Table S1. The results indicated that the candidate reference genes evaluated in this study encompassed a wide range of *C*_t_ values, ranging from 8 to 30, with the majority ranging from 16 to 27 (Figure 2, Supplementary Table S1). The *18S* gene was the most abundant reference gene in the tested Sacha inchi tissues with the lowest mean *C*_t_ value of 9, whereas *RPS13* was the least abundant reference gene with the highest mean *C*_t_ value of 25. The results also revealed that the *PLA* gene was characterized by the smallest variation in transcript levels among plant tissues, whereas the *GAPDH* gene displayed the largest variation among tissues.

The expression profiles of twelve candidate reference genes in various tissues are displayed in Supplementary Table S1. The *18S* gene was stably expressed in most tissues with high levels except in adult young leaves, and much lower levels in seeds at 90 and 130 days after pollination (DAP). *RPII* had relatively high abundance in young tissues. The expression of *EF1α*, *RPS13* and *UCE* varied during seed development. Across all the tissues tested, *GAPDH* and *TUB* had obvious expression variation, whereas *ACT*, *CYC*, *PLA*, *UBL* and *TEF2* had relatively stable expression.

### 2.3. Ranking of Candidate Reference Genes and Determination of the Optimal Reference Genes

In this study, to perform an all-sided analysis, the twelve candidate reference genes were evaluated in five experimental sets comprising samples collected at defined developmental stages. The first experimental set consisted of roots, stems, young leaves and mature leaves from three-week-old seedlings. The second set consisted of roots, stems, young leaves, mature leaves, young inflorescences and seeds (90 DAP) from one-year-old adult plants. The developmental stages of the flower (inflorescence buds and young inflorescences, female and male flowers) and seed (15, 40, 90 and 130 DAP) were included in the third and fourth experimental sets, respectively. The entire life cycle of Sacha inchi was analyzed in the fifth experimental set comprising all 16 samples described above. To obtain higher-accuracy stability rankings, four statistical algorithms (Δ*C*_t_ method, BestKeeper, NormFinder and geNorm) were applied to assess the *C*_t_ values for each candidate reference gene. The result indicated that the most appropriate reference genes differed among these statistical algorithms, whereas the identities of the inappropriate reference genes were largely consistent among the tested algorithms (Table 2 and Supplementary Table S2). The RefFinder, a web-based comprehensive tool integrating the above mentioned four computational programs (Available online: http://www.leonxie.com/referencegene.php), was also employed to calculate the recommended comprehensive ranking order. For the first experimental set (three-week-old seedlings), *UCE* and *ACT* were the two most stable reference genes based on the Δ*C*_t_, NormFinder and geNorm analyses, whereas *PLA* and *18S* were the two best reference genes based on the BestKeeper. According to the calculation performed by RefFinder, *UCE*, *ACT* and *PLA* were the three most stable reference genes in the seedling of Sacha inchi, whereas *GAPDH*, *TUB* and *UBL* were the least stable genes (Table 2).

For the adult plant set (Table 2), *RPS13* and *CYC* were the most stable reference genes according to the recommendations of Δ*C*_t_ and geNorm algorithms, *UCE* and *PLA* were recommended by BestKeeper, and *EF1α*, *ACT* and *RPII* were recommended by NormFinder. The comprehensive ranking order indicated that *RPS13*, *CYC* and *EF1α* were the three most stably expressed reference genes. The *18S*, *GAPDH* and *TUB* genes clearly showed the most variable expression levels.

During flower development, *PLA* and *UCE* were identified to be the most stable reference genes by Δ*C*_t_ and NormFinder, whereas *ACT*, *PLA* and *GAPDH* were identified by BestKeeper and geNorm. The comprehensive ranking order suggests that the *PLA*, *ACT* and *UCE* genes were the optimal reference genes and that the *18S*, *RPS13* and *TUB* genes were the least appropriate reference genes (Table 2). During seed development, *UCE*, *RPS13* and *EF1α* were the most appropriate reference genes according to the recommendations of Δ*C*_t_ and NormFinder; by contrast, *UBL*, *TEF2* and *PLA* were recommended by the BestKeeper algorithm, and the combination of *RPS13* and *RPII* were recommended by geNorm. The comprehensive ranking order suggests that *UCE*, *RPS13* and *RPII* were the optimal reference genes and that *18S*, *TUB* and *GAPDH* were the least appropriate reference genes (Table 2).

For the entire growth cycle of Sacha inchi, the two most stable reference genes based on the Δ*C*_t_, BestKeeper, NormFinder and geNorm algorithms were *UCE* and *ACT*, *PLA* and *UBL*, *ACT* and *EF1α*, and the combination of *ACT* and *UCE*, respectively. The comprehensive ranking order showed that the top three most stable reference genes were *UCE*, *ACT* and *EF1α* (Table 2).

**Table 2:** Stability ranking of candidate reference genes in different developmental stages.

Analysis Tool	Ranking Order (The 1st is the most stable, and the 12th is the least stable)											
	1	2	3	4	5	6	7	8	9	10	11	12
**Seedling**												
Δ*C*_t_	*UCE*	*ACT*	*CYC*	*PLA*	*18S*	*RPS13*	*EF1α*	*TEF2*	*RPII*	*UBL*	*TUB*	*GAPDH*
BestKeeper	*PLA*	*18S*	*TEF2*	*ACT*	*UCE*	*CYC*	*UBL*	*RPS13*	*RPII*	*EF1α*	*TUB*	*GAPDH*
NormFinder	*UCE*	*ACT*	*EF1α*	*RPS13*	*CYC*	*RPII*	*PLA*	*TUB*	*18S*	*TEF2*	*UBL*	*GAPDH*
geNorm	*ACT* | *UCE*		*PLA*	*18S*	*TEF2*	*CYC*	*RPS13*	*UBL*	*EF1α*	*RPII*	*TUB*	*GAPDH*
Recommended comprehensive ranking	*UCE*	*ACT*	*PLA*	*18S*	*CYC*	*TEF2*	*RPS13*	*EF1α*	*RPII*	*UBL*	*TUB*	*GAPDH*
**Adult Plant**												
Δ*C*_t_	*RPS13*	*CYC*	*EF1α*	*RPII*	*UCE*	*ACT*	*UBL*	*TEF2*	*PLA*	*TUB*	*GAPDH*	*18S*
BestKeeper	*UCE*	*PLA*	*UBL*	*CYC*	*TEF2*	*RPS13*	*ACT*	*RPII*	*EF1α*	*TUB*	*GAPDH*	*18S*
NormFinder	*EF1α*	*ACT*	*RPII*	*RPS13*	*CYC*	*UCE*	*PLA*	*UBL*	*TEF2*	*TUB*	*GAPDH*	*18S*
geNorm	*CYC* | *RPS13*		*EF1α*	*RPII*	*UCE*	*ACT*	*UBL*	*TEF2*	*PLA*	*TUB*	*GAPDH*	*18S*
Recommended comprehensive ranking	*RPS13*	*CYC*	*EF1α*	*UCE*	*RPII*	*ACT*	*PLA*	*UBL*	*TEF2*	*TUB*	*GAPDH*	*18S*
**Flower** **Development**												
Δ*C*_t_	*PLA*	*UCE*	*ACT*	*GAPDH*	*TEF2*	*EF1α*	*CYC*	*RPII*	*UBL*	*18S*	*RPS13*	*TUB*
BestKeeper	*ACT*	*PLA*	*GAPDH*	*UCE*	*TEF2*	*18S*	*CYC*	*UBL*	*EF1α*	*RPII*	*RPS13*	*TUB*
NormFinder	*UCE*	*PLA*	*TEF2*	*EF1α*	*ACT*	*GAPDH*	*RPII*	*CYC*	*RPS13*	*UBL*	*18S*	*TUB*
geNorm	*ACT* | *GAPDH*		*PLA*	*UCE*	*TEF2*	*CYC*	*UBL*	*EF1α*	*18S*	*RPII*	*RPS13*	*TUB*
Recommended comprehensive ranking	*PLA*	*ACT*	*UCE*	*GAPDH*	*TEF2*	*EF1α*	*CYC*	*UBL*	*RPII*	*18S*	*RPS13*	*TUB*
**Seed Development**												
Δ*C*_t_	*UCE*	*RPS13*	*EF1α*	*ACT*	*RPII*	*CYC*	*UBL*	*PLA*	*TEF2*	*GAPDH*	*TUB*	*18S*
BestKeeper	*UBL*	*TEF2*	*PLA*	*CYC*	*ACT*	*RPII*	*RPS13*	*UCE*	*EF1α*	*GAPDH*	*TUB*	*18S*
NormFinder	*UCE*	*EF1α*	*RPS13*	*RPII*	*ACT*	*CYC*	*UBL*	*PLA*	*GAPDH*	*TEF2*	*TUB*	*18S*
geNorm	*RPII* | *RPS13*		*EF1α*	*UCE*	*ACT*	*CYC*	*UBL*	*PLA*	*TEF2*	*GAPDH*	*TUB*	*18S*
Recommended comprehensive ranking	*UCE*	*RPS13*	*RPII*	*EF1α*	*UBL*	*ACT*	*CYC*	*PLA*	*TEF2*	*GAPDH*	*TUB*	*18S*
**Entire Growth Cycle**																									
Δ*C*_t_	*UCE*	*ACT*	*EF1α*	*CYC*	*RPII*	*RPS13*	*PLA*	*UBL*	*TEF2*	*TUB*	*18S*	*GAPDH*
BestKeeper	*PLA*	*UBL*	*TEF2*	*UCE*	*CYC*	*ACT*	*RPS13*	*RPII*	*EF1α*	*TUB*	*18S*	*GAPDH*
NormFinder	*EF1α*	*ACT*	*UCE*	*RPII*	*CYC*	*RPS13*	*PLA*	*UBL*	*TEF2*	*TUB*	*18S*	*GAPDH*
geNorm	*ACT* | *UCE*		*CYC*	*EF1α*	*RPII*	*RPS13*	*UBL*	*PLA*	*TEF2*	*TUB*	*18S*	*GAPDH*
Recommended comprehensive ranking	*UCE*	*ACT*	*EF1α*	*CYC*	*PLA*	*RPII*	*UBL*	*RPS13*	*TEF2*	*TUB*	*18S*	*GAPDH*

### 2.4. Reference Gene Validation

**Figure 3:**
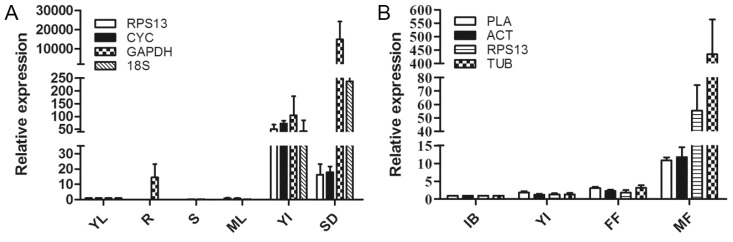
Relative quantification of the *AGAMOUS* homolog (*PvoAG*) in Sacha inchi using the validated reference genes for normalization. (**A**) *PvoAG* expression pattern in adult plants. The four kinds of bars indicate *PvoAG* expression levels normalized by different reference genes *RPS13*, *CYC*, *GAPDH* and *18S* respectively; and (**B**) *PvoAG* expression pattern during flower development. The four kinds of bars indicate *PvoAG* expression levels normalized by different reference genes *PLA*, *ACT*, *RPS13* and *TUB* respectively. YL, young leaf; R, root; S, stem; ML, mature leaf; YI, young inflorescence; SD, seed (90 DAP); IB, inflorescence bud; FF, female flower; MF, male flower.

Sacha inchi *AGAMOUS* (*PvoAG*, GenBank GADC01013770), with homologs in other plants that are mainly expressed in floral organs [29,30,31], was chosen to further validate the reliability of the selected reference genes for the normalization of RT-qPCR data in Sacha inchi adult plants and flower developmental stages. The most stable reference genes identified for adult plants (*RPS13* and *CYC*) and during flower development (*PLA* and *ACT*) were used as internal controls for data normalization. For comparison, the least stable reference genes identified in adult plants (*GAPDH* and *18S*) and during flower development (*RPS13* and *TUB*) were also considered. The results demonstrated that the expression patterns of *PvoAG* differed when using the most and least stable reference genes for normalization (Figure 3). In adult plants (Figure 3A), when the *RPS13* and *CYC* genes were used for normalization, *PvoAG* was predominantly expressed in young inflorescences with relatively lower expression in seeds (90 DAP). However, the expression level of *PvoAG* in seeds (90 DAP) was substantially greater than in young inflorescences when using the least stable reference genes (*GAPDH* and *18S*) for normalization. The *PvoAG* gene was also found to be expressed in mature roots when using *GAPDH* for normalization. During flower development (Figure 3B), when *PLA* and *ACT* were used for normalization, *PvoAG* was expressed in all developmental stages and at a higher level in male flowers. When *RPS13* and *TUB* were considered, the expression pattern of *PvoAG* was similar to that obtained when using the most stable reference genes, but the expression level was over-estimated in male flowers. These findings suggest that the choice of reliable reference genes is essential for the accurate normalization of target gene expression levels.

## 3. Discussion

As a result of its high sensitivity, specificity and cost-efficiency, RT-qPCR has greatly improved the quality of measurements of expression levels of target genes in biological samples [32]. However, the accuracy of RT-qPCR analysis can be significantly affected by several factors, including RNA quality, the quantity of cDNA and the selection of reference genes [9,16]. To achieve high accuracy, a reference gene should have a relatively stable expression level in distinct biological samples, such as across tissues, developmental stages and experimental conditions. In this study, the expression stabilities of twelve candidate reference genes were estimated in various tissues and developmental stages of Sacha inchi. The *UCE*, *ACT*, and *PLA* genes were found to be the most stable genes in seedlings. For roots, stems, leaves, flowers, and seeds from adult plants, *RPS13*, *CYC*, and *EF1α* were recommended as reference genes for RT-qPCR. During the development of reproductive organs, *PLA*, *ACT*, and *UCE* were the optimal reference genes for flower development, whereas *UCE*, *RPS13*, and *RPII* were optimal for seed development.

In this study, four computational methods (Δ*C*_t_, BestKeeper, NormFinder and geNorm) were used to evaluate the stability of the expression levels of these twelve candidate reference genes. Here we found that the least stable genes computed by the four algorithms were almost the same, while the most stable genes differed. In the set of adult plant, the *18S* gene was ranked last by all four algorithms, whereas the *RPS13*, the *UCE*, the *EF1α* and the combination of *CYC* and *RPS13* genes were ranked first by Δ*C*_t_, BestKeeper, Normfiner and geNorm, respectively (Table 2). To obtain the most stable reference gene, we used the RefFinder tool that integrates the currently available major computational programs (Δ*C*_t_, BestKeeper, Normfinder and geNorm) to compare and rank the tested candidate reference genes. Based on the rankings from each above mentioned program, RefFinder assigned an appropriate weight to an individual gene and calculated the geometric mean of their weights for the overall final ranking. Accordingly, the *RPS13* was recommended as the most appropriate reference gene in adult plant (Table 2).

The 18S ribosomal RNA is a component of the small subunit of eukaryotic ribosomes (40S). The *18S* gene has been used as a reference gene for RT-qPCR normalization in many previous studies [33,34]. In *Jatropha*, the *18S* was applied to normalize the expression of *JcAOC* and *JcBD1* in various tissues under salt and cold stress conditions [35,36]. However, in this study, the *18S* gene was the least stable gene across three experimental datasets, *i.e.*, the adult plant, the seed developmental stage, and the entire growth cycle of Sacha inchi. The *18S* gene has also been deemed inappropriate for gene expression analyses in *Pisum sativum* [37] and bamboo [20]. The *GAPDH* gene, which encodes a key enzyme involved in the glycolysis and gluconeogenesis [38], is another commonly used reference gene. It is the most stable reference gene in *Jatropha* over different tissues, developmental stages and experimental conditions [21]. And it has been also recommended in flax [39] and coffee [22,40]. However, *GAPDH* has been reported as the least stable reference gene in oil palm [23], peach [24], Petunia hybrida [25] and bamboo [20]. Similarly, in the present study, the *GAPDH* expression varied among tissues in Sacha inchi, except across the flower developmental stages in which it ranked the fourth. It is possible that *GAPDH* is not only a key enzyme involved in glycolysis but also participates in other processes. The *TUB* gene, which plays a crucial role in cell structural maintenance, has also been widely used as a reliable reference gene in switchgrass [41] and peach [24]. However, in our study, *TUB* was identified as a poor reference gene, similar to results for potato [42] and soybean [43]. Taken together, these results indicate that the most stable reference genes differ among plants or tissues. Hence, the choice of reference genes is very important.

Our results indicate that *ACT* is suitable for normalization in seedlings and during flower development in Sacha inchi. In *Jatropha*, *ACT* expression was more ubiquitous than in Sacha inchi, and was found across the different plant developmental stages and under cold-/drought-induced conditions [21]. *UCE* was ranked among the top three most stable reference genes for all tissues, with the exception that it was ranked fourth for the adult plant. Therefore, *UCE* is recommended for the normalization of gene expression in Sacha inchi. *UCE* has also been identified as one of the most stable reference genes in switchgrass [41], whereas *UCE* was the most variable reference gene for the tung tree [12] and *Jatropha* *curcas* [21]. In addition, we found that *CYC* was ranked first for the entire growth cycle of Sacha inchi and second for the adult plant in this study. The *CYC* gene was among the best reference genes for *Petunia hybrida* [37] and bamboo [24]. The *RPS13* gene, which was used for the normalization of gene expression in *Petunia hybrida* [25], might also serve as a reliable reference gene for studies of adult plants, different developmental stages of seeds, and the entire growth cycle of Sacha inchi.

To illustrate the actual utility of validated reference genes in this study, the expression pattern of *PvoAG* was examined in Sacha inchi. *AG* belongs to the C-class genes in the ABC model of floral organ development [44]. In *Arabidopsis*, *AG* was mainly expressed in inflorescences and flowers, and was involved in the regulation of stamen and pistil development [44]. In poplar and strawberry, *AG* was also highly expressed in flowers with low levels in leaves, stems and seeds [36,45]. Here, in Sacha inchi adult plants, *PvoAG* was remarkably expressed in young inflorescences with relatively lower expression in seeds (90 DAP) when the most stable genes *RPS13* and *CYC* were used for normalization (Figure 3A). This result is similar to the *AG* expressions in other plants mentioned above. However, when the least stable genes *GAPDH* and *18S* were applied, the expression level of *PvoAG* was extremely high in seeds (90 DAP) (Figure 3A). Thus, these results further proved the necessity of selection of reliable reference genes in gene expression studies.

To date, numerous studies have reported that when evaluating levels of target gene expression, the results are more pronounced and reliable when two or more reference genes are utilized [42,46,47]. In this study, we have recommended the three most reliable reference genes for expression analyses of Sacha inchi for each of the aforementioned experimental conditions. The results of this study will help inform the selection of stable reference genes for future gene expression studies of Sacha inchi.

## 4. Experimental Section

### 4.1. Plant Materials

During the vegetative growth stage, tissues (roots, stems, young leaves and mature leaves) were collected from Sacha inchi (*Plukenetia volubilis* L.) seedlings that were grown in a growth chamber for three weeks after germination (12 h light/day, 25 °C). During the reproductive growth stage, tissues (roots, stems, young leaves, mature leaves, inflorescence buds, young inflorescences, female flowers, male flowers, and seeds at 15, 40, 90 and 130 DAP, respectively) were collected from one-year-old adult plants of Sacha inchi, which were grown in a field at the Xishuangbanna Tropical Botanical Garden (XTBG, 21°54′N, 101°46′E, 580 m in altitude) of the Chinese Academy of Sciences located in Mengla County, Yunnan Province, Southwest China [48]. The reproductive organs are shown in (Supplementary Figure S3). All of the tissues removed from plants were immediately frozen in liquid nitrogen and stored at −80 °C. Three biological replicates were collected for each sample.

### 4.2. Total RNA Extraction and cDNA Synthesis

Total RNA was isolated using the pBIOZOL Plant Total RNA Extraction Reagent according to the manufacturer’s instructions (BioFlux, Hangzhou, China). The RNA integrity was evaluated on a 2% agarose gel. The quantity and quality of the total RNA samples were assessed by measuring the absorbance ratio at 260/280 and 260/230 nm using a NanoDrop ND-1000 spectrophotometer (Thermo Scientific, Wilmington, DE, USA). Total RNA samples with A_260_/A_280_ and A_260_/A_230_ ratios greater than 1.8 were used for cDNA synthesis. An aliquot of total RNA (1 μg) was reverse transcribed using the PrimeScript™ RT reagent Kit with gDNA Eraser in a 20-μL reaction volume according to the manufacturer’s protocol (Perfect Real Time). All of the cDNA samples were diluted at 1:5 with RNase-free water and stored at −80 °C.

### 4.3. Selection of Candidate Reference Genes and Design of RT-qPCR Primers

Twelve Sacha inchi housekeeping genes (*18S*, *ACT*, *CYC*, *EF1α*, *GAPDH*, *PLA*, *RPII*, *RPS13*, *TEF2*, *TUB*, *UBL* and *UCE*) were selected as candidate reference genes. The cDNA sequences of these reference genes (Supplementary Figure S1) were obtained from the GenBank database (Available online: http://www.ncbi.nlm.nih.gov/nucleotide) and our RNA-seq transcriptome dataset of Sacha inchi. RT-qPCR primers (Table 1) were designed using Primer Premier 6 software [49] with the following parameters: melting temperature between 59 and 61 °C, primer length of 22–27 nucleotides, GC content of 40% to 60%, and PCR amplicon length of 101–202 bp.

### 4.4. RT-qPCR Conditions and Data Analysis

RT-qPCR was performed in a 96-well plate with a Roche LightCycler 480 real-time PCR detection system (Roche Diagnostics, Rotkreuz, Switzerland). The reaction was performed in a volume of 20 μL containing 1 μL of diluted cDNA, 10 μL of SYBR Premix Ex Taq™ II (Tli RNaseH Plus), and 0.25 μM of each primer. For each reference gene, no-template reactions were run as negative PCR controls. The cycling conditions were as follows: initial activation of 5 min at 95 °C; 45 cycles of 10 s at 95 °C, 20 s at 59 °C (60 °C for *UCE*); and 20 s at 72 °C. The specificity of the PCR amplicons was verified based on the melting curve from 60 to 95 °C. Each reaction was performed in three technical replicates with three biological replicates for each tissue. To calculate the gene-specific PCR efficiency, standard curves were generated from 10-fold serial dilutions of cDNA samples from young leaves for each primer pair. The values of the slopes and correlation coefficients were obtained from the standard curves. The corresponding PCR amplification efficiencies (E) were calculated according to the equation E = −1 + 10 ^(−1/sl^^o^^pe)^ [50].

Gene expression stability was evaluated by applying four statistical algorithms: Δ*C*_t_ [14], geNorm (version 3.5) [15], BestKeeper (version 1.0) [16], and NormFinder (version 0.953) [17]. The RT-qPCR data obtained from the Roche LightCycler 480 manager were exported into an Excel datasheet. Each statistical algorithm generates a measurement of reference gene stability that can be used to rank the stability order using RefFinder (Available online: http://omictools.com/reffinder-s2857.html) [51].

## 5. Conclusions

Twelve reference genes were evaluated in multiple tissues and during multiple developmental stages of flowers and seeds in Sacha inchi. The *UCE*, *ACT* and *PLA* genes were the most stable reference genes for seedlings of Sacha inchi, whereas the *RPS13*, *CYC* and *EF1α* genes were the most suitable reference genes for adult plants. The *PLA*, *ACT* and *UCE* genes are recommended as reference genes during flower development, and the *UCE*, *RPS13* and *RPII* genes are recommended for studies during seed development. For analyses of the entire growth cycle of Sacha inchi, the three best reference genes are *CYC*, *RPS13* and *UCE*.

## Supplementary Materials

Supplementary materials can be found at http://www.mdpi.com/1422-0067/16/06/12513/s1.
