# Supplementary material for: Selection of Reliable Reference Genes for Gene Expression Studies of a Promising Oilseed Crop, Plukenetia volubilis, by Real-Time Quantitative PCR
Source: Int J Mol Sci. 2015 Jun 3;16(6):12513–30. doi: 10.3390/ijms160612513 (PMC4490458; doi:10.3390/ijms160612513)
Supplement: Supplementary file 1 [file ijms-16-12513-s001.pdf]

Supplementary Information

>Pvo18S RNA (5′–3′)
TAGTTTGTTTGATGGTACCTACTACTCGGATAACCGTAGTAATTCTAGAGCTAATACGTGCAACAAACCCCGACTTCTGGAAGGGAT
GCATTTATTAGATAAAAGGTCGACGCGGGCTCTGCCCGTCGCTCTGACGATTCATGATAACTCGACGGATCGCACGGCCTTCGTGCC
GGCGACGCATCATTCAAATTTCTGCCCTATCAACTTTCGATGGTAGGATAGAGGCCTACCATGGTGGTGACGGGTGACGGAGAATTA
GGGTTCGATTCCGGAGAGGGAGCCTGAGAAACGGCTACCACATCCAAGGAAGGCAGCAGGCGCGCAAATTACCCAATCCTGACA
CGGGGAGGTAGTGACAATAAATAACAATACCGGGCTCATCGAGTCTGGTAATTGGAATGAGTACAATCTAAATCCCTTAACGAGGAT
CCATTGGAGGGCAAGTCTGGTGCCAGCAGCCGCGGTAATTCCAGCTCCAATAGCGTATATTTAAGTTGTTGCAGTTAAAAAGCTCG
TAGTTGGACCTTGGGATGGGTCGTCCGGTCCGCCTAGCGGTGTGCACCTGTCGACTCGTCCCTTTTGCCGGCGATGCGCTCCTGGC
CTTAACTGGCCGGGTCGTGCCTCCGGCGCTGTTACTTTGAAGAAATTAGAGTGCTCAAAGCAAGCCTACGCTCTGTATACATTAGC
ATGGGATAACATCATAGGATTTCGGTCCTATTCTGTTGGCCTTCGGGATCGGAGTAATGATTAACAGGGACAGTCGGGGGCATTCGT
ATTTCATAGTCAGAGGTGAAATTCTTGGATTTATGAAAGACGAACAACTGCGAAAGCATTTGCCAAGGATGTTTTCATTAATCAAGA
ACGAAAGTTGGGGGCTCGAAGACGATCAGATACCGTCCTAGTCTCAACCATAAACGATGCCGACCAGGGATCGGCGGATGTTGCTT
TTAGGACTCCGCCGGCACCTTATGAGAAATCAAAGTCTTTGGGTTCCGGGGGGAGTATGGTCGCAAGGCTGAAACTTAAAGGAATT
GACGGAAGGGCACCACCAGGAGTGGAGCCTGCGGCTTAATTTGACTCAACACGGGGAAACTTACCAGGTCCAGACATAGTAAGG
ATTGACAGACTGAGAGCTCTTTCTTGATTCTATGGGTGGTGGTGCATGGCCGTTCTTAGTTGGTGGAGCGATTTGTCTGGTTAATTC
CGTTAACGAACGAGACCTCAGCCTGCTAACTAGCTATGCGGAGGTATCCCTCCGCGGCCAGCTTCTTAGAGGGACTATGGCCTTCT
AGGCCAAGGAAGTTTGAGGCAATAACAGGTCTGTGATGCCCTTAGATGTTCTGGGCCGCACGCGCGCTACACTGATGTATTCAACG
AGTCTATAGCCTTGGCCGACAGGCCCGGGTAATCTTTGAAATTTCATCGTGATGGGGATAGATCATTGCAATTGTTGGTCTTCAACG
AGGAATTCCTAGTAAGCGCGAGTCATCAGCTCGCGTTGACTACGTCCCTGCCCTTTGTACACACCGCCCGTCGCTCCTACCGATTGA
ATGGTCCGGTGAAGTGTTCGGATCGCGGCGACGTGGGCGGTTCGCTGCCGGCGACGTCGCGAGAAGTCCACTGAACCTTATCATTT
AGAGGAAGGAGAAGTCGTAACAAGGTTTCCGTAGGTGAACCTGCGGAAGGATCATTGTCGA

>PvoACT (5′–3′)
ATGGCTGATGGTGAAGATATTCAGCCCCTCGTTTGTGACAATGGAACTGGAATGGTGAAGGCTGGGTTCGCCGGTGATGATGCTCC
AAGGGCAGTGTTCCCTAGCATTGTTGGAAGACCTAGACATACTGGCGTCATGGTTGGGATGGGTCAGAAAGATGCTTATGTAGGTG
ATGAAGCACAGTCTAAAAGAGGTATCCTGACCTTGAAATACCCCATTGAGCATGGTATAGTTAGCAATTGGGATGATATGGAAAAGA
TCTGGCATCATACTTTTTACAATGAGCTCCGTGTTGCACCTGAGGAGCATCCAGTGCTTCTCACTGAGGCCCCTCTTAACCCTAAGG
CCAACAGAGAGAAGATGACTCAGATCATGTTTGAGACCTTTAATGTCCCTGCAATGTACGTCGCCATTCAGGCTGTTCTCTCCTTGT
ATGCCAGTGGCCGTACAACTGGTATCGTGCTTGATTCTGGTGATGGTGTGAGTCACACTGTGCCAATTTATGAAGGTTACGCTCTCC
CACATGCCATCCTCCGTCTGGACCTTGCTGGACGTGATCTCACTGATGCTTTAATGAAGATTCTCACTGAGAGAGGGTACATGTTCA
CCACCACTGCTGAACGGGAAATTGTCCGTGACATGAAGGAGAAACTTGCATATGTTGCCCTTGACTATGAGCAGGAGCTTGAGACT
GCCAAGAGCAGCTCCTCTGTTGAAAAGAACTACGAGCTTCCTGATGGCCAGGTCATCACCATTGGAGCCGAGAGATTCCGTTGCC
CAGAAGTCTTGTTCCAGCCATCTCTCATTGGAATGGAAGCCGCAGGCATTCATGAAACTACTTACAACTCTATCATGAAGTGTGATG
TCGATATTAGGAAGGACCTTTATGGTAATATTGTGCTTAGTGGTGGTTCCACTATGTTCCCTGGTATTGCAGACCGTATGAGCAAGGA
GATCACCGCGCTTGCTCCCAGCAGTATGAAGATTAAGGTGGTGGCTCCACCAGAGAGAAAATACAGTGTCTGGATTGGAGGATCTA
TCCTTGCATCCCTCAGTACCTTCCAGCAGATGTGGATTTCCAAGGGCGAGTATGACGAGTCTGGTCCATCCATTGTCCACAGGAAGT
GCTTC

Figure S1. *Cont.*

PvoCYC (5′–3′)

GGTCGCATAGTCATGGGTCTCTTTGGCAACACAGTTCCTAAGACTGCAGAAAATTTTCGAGCACTTTGTACGGGAGAGAAAGGAAT
TGGAAAAAATGGGAAGCCTCTTCATTACAAAGGAAGCTCTTTCCACAGGATTATTCCCAGCTTTATGATTCAAGGTGGTGATTTTAC
CCGAGGTGATGGACGAGGTGGAGAGTCAATCTATGGTGAGAAGTTCGCCGATGAAAATTTCAAGCTGAAGCACACTGGACCAGGA
ATTCTGTCAATGGCAAATGCTGGGCAAGATACGAACGGATCACAGTTCTTTGTAACTACTGTAATGACCAGCTGGTTGGATGGCCAT
CACGTGGTGTTCGGCAAGGTGCTTTCTGGGATGGATGTTGTCTACAAGATTGAAGCTGAAGGAAGTCGGAGTGGAGTGCCTAGAA
GAAAAGTTGTCATTTTAGACAGTGGTGAATTGCCTTTA

>PvoEF1α (5′–3′)

GCGACCACCCCCAAGTACTCTAAGGCCAGGTATGATGAAATTGTCAAGGAAGTTTCTTCATACCTTAAGAAGGTTGGATACAACCC
TGAAAAGATCCCATTTGTTCCTATTTCTGGTTTCGAAGGTGACAACATGATTGAGAGATCCACCAATCTGGATTGGTACAAGGGTCC
AACCTTGCTCGAGGCTTTAGACCTGATCAATGAGCCAAAGAGGCCCACAGATAAGCCTCTTCGTCTGCCACTCCAGGACGTTTACA
AGATTGGTGGTATTGGAACTGTCCCAGTGGGTCGTGTGGAAACAGGTATTCTCAAGCCTGGTATGGTTGTGACCTTTGGACCAAGT
GGGCTAACAACTGAAGTTAAGTCTGTTGAGATGCATCATGAGGCTCTTCAGGAGGCTCTCCCTGGTGACAACGTTGGCTTCAACGT
TAAGAATGTTGCTGTCAAGGATCTGAAGAGAGGATACGTTGCATCCAACTCAAAGGATGATCCTACCAAGGAGGCTGCAAACTTCA
CATCCCAGGTTATCATCATGAACCACCCTGGTCAGATTGGTAACGGATATGCCCCAGTACTGGACTGCCACACCTCCCACATTGCTG
TGAAGTTTGCCGAGATCCTGACCAAGATTGACCGTCGATCTGGAAAAGAGCTTGAAAAGGAACCCAAATTCTTGAAGAATGGTGA
TGCTGGTTTCGTGAAGATGATTCCAACAAAGCCCATGGTGGTTGAAACCTTCTCCGAGTATCCTCCACTGGGTCGTTTTGCTGTCAG
GGACATGCGTCAGACTGTTGCCGTCGGTGTCATCAAGAGTGTTGAGAAGAAGGATCCATCTGGAGCCAAGGTCACCAAGTCCGCT
GTCAAGAAGGGAGGAAAA

>PvoGAPDH (5′–3′)

ATGGCTTCGGCTACTTTCTTTGTAGCCAAACCATCCCTTCAGGGAAATGGAAAAGGATTCCAGGAATTTTCAGGACTCCGTAACTC
ATCAGCTTTCCCTTCTTTTTCCAGGAAATCTTCTGATGACTTCCTCTCTGTCGTTGCTTTCCAGACTTCTGCTGTTGGAAGCAGCAAT
GTAGGATACAGGAAAATTGCAGCTATGGCAAAGCTAAAAGTTGCCATTAATGGATTTGGAAGGATTGGAAGGAACTTCTTGAGGTG
CTGGCATGGACGCAAAGATTCCCCACTTGATGTCATTGCCATCAACGACAGTGGCGGTGTCAAGCAAGCTTCTCACCTCCTGAAAT
ACGATTCTACGCTTGGCATCTTTGAAGCTGATGTCAAGCCTGTTGGTACTGATGCAATCTCTGTCGATGGCAAGGTCATCAAGGTTG
TTTCTGACAGAAACCCTGTCAACCTTCCATGGAAGGACTTGGGGATCGACCTGGTGATCGAAGGAACCGGAGTTTTCGTTGACAG
GGAAGGTGCTGGCAAGCATATTCAGGCAGGAGCTAAGAAGGTTCTCATCACAGCGCCTGGAAAGGGTGACATCCCAACCTATGTT
GTGGGAGTCAATGCTGATACCTACAATCCTGATGAGCCAATCATCAGCAATGCTTCTTGCACTACTAACTGCCTTGCTCCCTTTGTC
AAGGTCCTTGACCAGAAATTTGGGATCATCAAGGGTACCATGACAACAACTCACTCGTACACCGGCGACCAAAGGCTGCTCGACG
CGAGCCATCGTGACTTAAGAAGAGCAAGAGCCGCCGCTCTAAATATTGTTCCAACATCAACAGGCGCAGCCAAGGCAGTAGCCCT
CGTCCTTCCATCTCTGAAAGGCAAACTAAATGGCATCGCATTGCGAGTTCCAACTCCAAACGTATCAGTCGTCGATCTCGTAATCCA
GGTGGAGAAGAAGACCTTTGCAGAAGAAGTAAACGCGGCGTTTAGAGAGAGTGCAGAGAATGAACTAAAGGGTATACTGTCAGT
CTGCGACGAGCCACTTGTTTCGGTTGATTTCAGGTGCACAGATGTTTCTTCGACAGTTGATTCTTCGTTGACAATGGTGATGGGAG
ATGACATGGTTAAGGTCATTGCTTGGTATGATAATGAATGGGGTTATTCTCAAAGGGTTGTGGATTTGGCTGACATTGTTGCCAGTAA
CTGGAAA

Figure S1. *Cont.*

>PvoPLA (5′–3′)
TTAGGTCACACCTTTGATATGCATTCGGATAAGGATTTGGGAGTGAGCCTTAAATTCAATGATGCTATTTTATTGAATACCATAGAAA
AGCAAGAGCAAACGTCTCATTTTGATTACAAGGTGTTGGGATGGTCTTTCCTTCCATTTGTACCTTCGGGTATCAATACTAAAGTTA
ATGTTCGAACACCGATTGCGATAAACAAAGGGTTTGAACATTCAGAGACTGATTTAGGAACAATTTTCAAACCCACAAATGCTGTT
TTGTCCAGGACTTCTACTGCCATAGAAAAACCAAAGCAGAAGATCCATTTTGATATCAAGTTCTTGGGATGGTCCCTCCTATCCTTG
TTACCTTGGACTATCAATGCTAATGATAGTATTCGAACACCGCCGGCTACCATAAACAGAGGGTTGAAAAGGCGTGCCCGATCTCGT
GGTGCCGTTGAAAATGGTGAATCTTTGCGCTTTAGACCATATGTCTCCAAGGTTCCTTGGCATACTGGTCCAAGAGGCTTTCTTTCT
CTGCTATTTCCACGTTACGGGCATTATTGTGGGCCCAACTGGTCAAGTGGAAAAGATGGTGGGTCTCCTGTTTGGGATAAGAGGCC
AATAGATTGGTTGGATTATTGCTGCTACTGCCATGACATGGGTTATGACACTGATGACCAAGCCAGGCTGCTGAAGGCCGATCTACA
GTTTCTGGAGTGCTTGGAGAGACCACGTGTTACTTCTAAAGGAGACACGGGTATTGCTTGTGTTTATAAAACAATGTGCATCACAG
GTATGCGGAGCAATACAATCTTTTTTCTATTATATGATAATTAGCCTATGAAATAAGTTTTTTCCTTCTTCAGATGGGATGAATGCAGG
TCTGTGTGCTTTCTCATGGTGCTTAAATTATAGGTTAGTCGTTGTGTTACTTTTTGTCTTAAATGTATTGTAGGGCTTAGGAACTTCCT
GATACCATACAGAACGCAGCTTGTGAACTTGGAGCTTAGGCCACCAATCCTTAATTTGGATGGCTGAGCAATGTTAAATGGATAGG
TTGGAACTGGCGGAAAACA

>PvoRPII (5′–3′)
ATGGAAGGAGCTTCCAAATACCAGCGTCTTCCAAGAATCAAAATCCGAGAACTGAAAGATGATTACTGCAAATTCGAACTGCGCG
ATACCGACGCTTCTATGGCAAACGCGTTGCGGCGCGTGATGATTTCCGAAGTTCCCACCATAGCTATCGACTTGGTCGAAATCGAAG
TTAACTCTTCGGTCTTAAACGACGAGTTTATTGCTCACCGCCTCGGTCTCATTCCTCTTACAAGCGAACGTGCTATGAGCATGCGCT
TCTCACGTGATTGTGATGCTTGTGATGGTGACGGTCAGTGCGAGTATTGTTCTGTTGAGTTTCATCTCCGTGCTAGATGTATCACTGA
TCAAACCCTAGATGTTACTAGTAAGGATTTGTATTCTTCTGATCATACTGTTGTTCCTGTTGATTTCTCCGATTCTGCTGCTTTTGACT
CCTCTGAGCAAAGGGGAATTACTATCGTCAAGCTGCGGAGAGGGCAAGAATTAAGATTGAGAGCAATTGCTAGGAAAGGAATTGG
GAAAGATCATGCGAAATGGTCGCCTGCGGCAACAGTGACATTCATGTATGAACCTGATATTCATATTAATGAGGATTTGATGGAAAC
TTTGTCGCTTGAGGAGAAACAAAGTTGGGTTGAAAGTAGTCCTACGAAAGTGTTTGACATTGATCCTAATACTCAACAGGTTGTAG
TTGTTGACCCTGAAGCATACACTTACGATGATGAAGTGCTCAAGAAAGCCGAAGCTTTGGGGAAATCGGGGCTCGTGGATATCCGT
GCAAAGGAAGACAGTTTTATATTCACGGTTGAATCAACTGGTGCAATTAAAGCTTCTCAGCTGGTACTCAATGCCATAGAAGTTCTA
AAACAAAAATTGGATGCTGTTCGCTTGTCTGAAGACACCGTGGAAGCTGATGATCAGTTTGGTGAGCTAGGTGCTCACATGCGCGG
AGGA

>PvoRPS13 (5′–3′)
AAAAATTTGTCGTTTCGTTGGCAGAGGGTACAATGTCTGCGTATCGGTAATGCACAGCTTCCAGATGACAAGAGATTGCGGTTTTCT
CTTCAGCACATCCGTGGAATTGGGCGCCAAAGAGCTCATCAAATCCTTGTTGACTTAAGCCTAGAGAACAAGCACACCAAGAACT
TGACTGGATTGGAACTCCACTCTCTTAGAGATGAAGTCTCCAAGTACTTGATTGGAGAAGAATCAAAGCGACTGGTTGGAAGTGAT
ATACAGAAATTGACAGACATTCAGTGCTACCGAGGATATAATCATGCTTCCTGTTTGCCTTGTAGAGGACAGCGTACTAAAACCAAT
GCTCAAACGAGGAAGGGACAAAGA

Figure S1. *Cont.*

>PvoTEF2 (5′–3′)

TTGATGGATTCGAAAGCGAGAGAAAAGCGTCTGCTAGAGTTGTTTCGAGTGGCATGGAATGGTGCCATAGGAGCTGCGCTTCATAG
AAAACAAGACGAAGATCGATGCCTCCATGCCTTATCTCGCTTAAAGTTCTTTCCTGTTTCCAAAGATATTCTTGGTTCGACCAATGT
CGCCATCAGCATCTATAATCTCCAGACCCATTCCAGCAACAGAATTCAATGTCTGTCTCGTGAAGTTATCACCGCATGGAAGAAGAA
GGTTCATGATCTCGTTTTGCGAACATCCTTCACGCCCATGAAACCTGGAGATGATTCAAGTAAAGCTGGAAACAGAGTTACCGTTC
CTTTGAATCGCGTCAAGATTCAGAGCATGAAGAGGGACAAGTTACAGGAGCAAATTCTCGAGGGTTTGCGCATGGTGTTGAAAGA
AGATTCATCTGCTTATGCATATGATGCAGCAAGAGTCGCATCTTCCCTGGAGTCGGAGTTGTTCTTGCAATGGGGTGCTCCTGCCGA
TGGAAATCGCAACAAATACCGATCGTTGTTGTTCAACATGAATGCATTGGAGATGGCTAGTGATGCGATGAAGCAGGATGTCAAAT
GTATTCAAGCCAAAGCTCTGGAGAAGTGT

>PvoTUB (5′–3′)

ATGCTCACATTCTCTGTGTTTCCATCGCCGAAAGTTTCGGACACGGTGGTTGAGCCTTATAATGCTACTCTTTCTGTTCATCAGTTGG
TTGAGAATGCCGATGAATGCATGGTTCTTGATAATGAGGCTCTCTACGATATCTGCTTCAGGACTCTTAAGCTCACCACTCCTAGCTT
TGGTGATTTGAACCACTTGATCTCTGCAACTATGAGTGGAGTAACATGCTGCCTGAGGTTCCCTGGTCAACTCAACTCTGACCTCC
GAAAGCTTGCTGTGAACCTTATCCCATTCCCTCGTCTTCACTTCTTCATGGTTGGTTTCGCTCCTTTGACATCTCGTGGTTCTCAGCA
ATACCGGGCATTAACAGTGCCAGAACTTACTCAGCAAATGTGGGATGCCAAGAACATGATGTGTGCTGCTGATCCTCGGCACGGGC
GCTACCTCACTGCATCTGCTATGTTCCGTGGAAAGATGAGCACCAAGGAAGTGGATGAGCAGATGATCAATGTTCAGAACAAGAA
CTCGTCCTACTTTGTGGAGTGGATTCCGAACAATGTGAAATCCAGTGTCTGTGACATTCCGCCTCGAGGGCTTTCCATGGCATCCAC
CTTCGTTGGGAACTCAACTTCCATCCAGGAAATGTTTAGGAGGGTAAGCGAACAATTCACTGCCATGTTCAGGAGAAAAGCTTTCT
TGCATTGGTACACGGGTGAAGGAATGGATGAGATGGAGTTCACCGAAGCTGAGAGCAATATGAATGATCTCGTGTCGGAGTACCAG
CAGTACCAGGATGCAACTGCTGATGACGAAGGTGACTACGAAGATGACGAGGAAGATGAATCGGTGCAT

>PvoUBL (5′–3′)

CCACCACGAAGGCGGAGGACAAGGTGCAGAGTGGATTCCTTTTGAATATTGTAGTCTGCCAATGTGCGTCCATCTTCCAATTGCT
TCCCAGCGAAGATCAGCCTTTGCTGGTCTGGGGGAATACCCTCCTTGTCCTGGATCTTAGCCTTCACGTTATCAATTGTATCTGAA
CTCTCAACCTCCAGCGTGATGGTTTTCCCAGTCAGAGTTTTGACGAATATCTGCATTCCTCCACGCAGACGTAGCACCAGATGCA
GAGTAGACTCCTTCTGAATGTTGTAGTCAGCAAGAGTCCTTCCATCTTCAAGCTGCTTACCAGCGAAAATCAACCTCTGCTGGTCC
GGTGGGATCCCTTCTTTATCTTGGATTTTGGCCTTGACATTATCAATGG

>PvoUCE (5′–3′)

ATGACGCTTGGCGGCTCAGGTGGATCTAGTGTCGTGGTTCCTCGAAACTTCAGATTGCTGGAGGAACTTGAACGTGGTGAAAAAG
GTATTGGAGATGGTACAGTAAGCTATGGAATGGATGACGGAGACGACATCTACATGCGTTCGTGGACTGGAACAATAATTGGTCCTC
ACAATACTGTGCATGAAAGTAGAATTTATCAGTTGAAGCTATTTTGTGATAAGGATTATCCAGAGAAGCCACCAAGTGTTCGATTTC
ATTCACGGATTAATATGACTTGTGTTAACCATGAAACTGGAGTGGTGGAACCAAAGAAGTTTGGACTTCTTGCAAACTGGCAGCGG
GATTACACGATGGAGGATATTCTGACACAGCTGAAGAAGGAGATGGCCACCCCACACAACCGGAAGCTAGTTCAACCTCCTGAGG
GTACCTACTTC

Figure S1. *Cont.*

>PvoAG (5′–3′)

CATGATACGCCAGCTTTTAGGTGACCTATAGAATACTCAAGCTATGCATCCAACGCGTTGGGAGCTCTCCCATATGGTCGACCTGCA
GGCGGCCGCGAATTCACTAGTGATTCGAGCTCCTTCCTTCCATTTTCTGCATCTCTTTCTCTAGTACTCAATCAGATTTATAGAAACC
CAAGAAAAAAAAAACAACTTTCTTTCTCTCCTCAATTAGCATCTTCATTTTGTTTATTTCCTATCAAAGATCAGCTGCCATGGCATAT
CAAGGCGATTCGGGGGAGAACTCACCTCAGAGGAAATTGGGTAGGGGAAAGATCGAGATCAAGCGGATCGAGAACACCACAAAT
CGCCAAGTTACTTTCTGCAAAAGAAGGAATGGTTTGCTGAAGAAAGCTTATGAACTGTCTGTTCTTTGTGATGCTGAAGTTGCTCT
TATTGTCTTCTCTAGCCGCGGCCGCCTCTATGAGTACGCTAACAATAGCGTTAAATCAACGATTGAGAGGTATAAAAAGGCTAGCTC
GGATTCATCCAACACTGGCTCCATCGCTGAAGCTAATGCCCAGTTCTATCAGCAAGAAGCGGCCAAACTACGTGTGCAAATTGGCA
ATTTGCAGAACTCAAACAGGCATATGATGGGGGAGTCGTTGGGAGGCTTGAACGTCAAAGACCTTAAGAGCCTGGAGATAAAATT
AGAAAAAGGAATTAGCAGGATTCGATCAAAGAAGAACGAGTTGTTGTTCGCAGAGATTGAGTATATGCAGAAAAGAGAAATTGAT
CTGCACAACAATAACCAGTTTCTTCGAGCCAAGATTGCTGAAAATGAAAGGAAGCAGAAGAACATGAATTTGATGCCAGTAGGTG
ATAACTATGAGATAATGCAACCTCATCCATATGATAACCGCAACTATTTTCAAGTGAATGCATTACAACCCACGAATCATTACCCGCC
TCAAGACCAAATGTCGCTTCAGTTAGTTTAAAGTCTGTCGTCTTTTCTCTCGATTTTCTGTGGGAAGAGAAGTTAATCAGGTATTTC
ACCAACTGAAAAGCTGCAAAAAAATCCTGAAAAACTTCTAGCAGAGAAATCAAAACCTGAGCAAATCCTTCCATTCATAATTACTA
ATGTGAAATCCAA

Figure S1. The cDNA sequences of the twelve candidate reference genes and *PvoAG* gene. Yellow highlighted sequences are forward primers sequences; green highlighted sequences are reverse primers sequences.

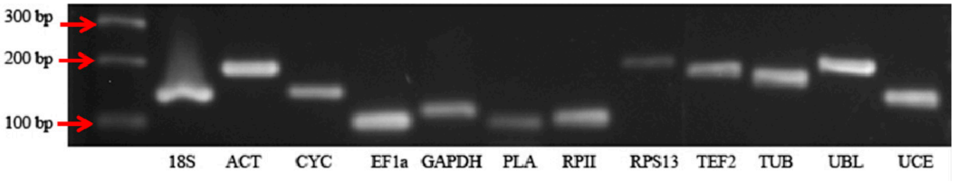

Figure S2. The PCR products electrophoresis result of the twelve candidate reference genes.

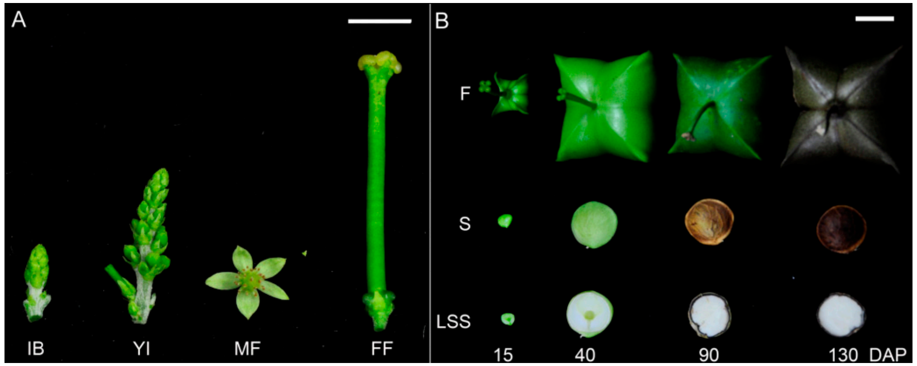

Figure S3. The appearance of Sacha inchi during various developmental stages of the reproductive organs. (**A**) Developmental stages of flower. IB, inflorescence bud; YI, young inflorescence; MF, male flower; FF, female flower. Bar = 0.5 cm; and (**B**) Developmental stages of fruit and seed. Fruit and seed from 15 to 130 DAP. F, fruit; S, seed; LSS, longitudinal section of seed; DAP, days after pollination. Bar = 1 cm. The PCR products electrophoresis result of the twelve candidate reference genes.

Table S1. Cycle threshold (C_t) values for twelve candidate reference genes.

Tissue	Genes and C_t Values											
**Samples**	***18S***	***ACT***	***CYC***	***EF1α***	***GAPDH***	***PLA***	***RPII***	***RPS13***	***TEF2***	***TUB***	***UBL***	***UCE***
SR	8.03 ± 0.13	17.59 ± 0.20	21.98 ± 0.11	19.48 ± 0.06	30.19 ± 0.22	23.35 ± 0.09	22.94 ± 0.04	25.89 ± 0.17	23.54 ± 0.08	24.28 ± 0.15	16.91 ± 0.12	21.90 ± 0.07
SS	8.06 ± 0.08	16.35 ± 0.19	21.43 ± 0.08	17.94 ± 0.04	19.61 ± 0.11	23.35 ± 0.19	22.22 ± 0.35	24.06 ± 0.22	23.98 ± 0.42	20.93 ± 0.04	16.97 ± 0.21	20.53 ± 0.04
SYL	8.03 ± 0.13	16.14 ± 0.03	20.56 ± 0.14	16.27 ± 0.11	18.01 ± 0.11	22.73 ± 0.17	19.73 ± 0.19	24.69 ± 0.13	23.70 ± 0.10	19.68 ± 0.08	17.34 ± 0.06	20.33 ± 0.08
SML	8.87 ± 0.03	17.01 ± 0.18	22.92 ± 0.14	19.17 ± 0.05	17.19 ± 0.05	23.70 ± 0.12	22.84 ± 0.05	26.25 ± 0.26	24.76 ± 0.21	22.40 ± 0.06	19.04 ± 0.14	21.30 ± 0.13
AR	10.91 ± 0.09	18.73 ± 0.22	23.63 ± 0.07	20.47 ± 0.11	27.56 ± 0.15	23.85 ± 0.07	24.31 ± 0.01	27.46 ± 0.15	25.05 ± 0.37	24.38 ± 0.15	19.62 ± 0.20	22.36 ± 0.04
AS	8.29 ± 0.15	15.44 ± 0.05	20.71 ± 0.14	16.83 ± 0.11	18.34 ± 0.52	23.58 ± 0.09	21.00 ± 0.13	24.39 ± 0.12	22.97 ± 0.32	19.39 ± 0.21	16.87 ± 0.23	20.87 ± 0.06
AYL	23.58 ± 0.18	19.46 ± 0.22	22.69 ± 0.04	20.94 ± 0.25	20.81 ± 0.10	24.26 ± 0.03	24.43 ± 0.15	26.80 ± 0.37	25.33 ± 0.11	21.34 ± 0.15	18.72 ± 0.06	22.61 ± 0.11
AML	9.36 ± 0.04	16.64 ± 0.07	22.47 ± 0.02	19.26 ± 0.04	17.29 ± 0.18	22.96 ± 0.03	22.80 ± 0.05	26.70 ± 0.02	23.53 ± 0.18	22.64 ± 0.22	17.63 ± 0.10	22.03 ± 0.15
IB	9.31 ± 0.07	16.97 ± 0.13	22.17 ± 0.08	17.28 ± 0.06	19.23 ± 0.28	22.71 ± 0.23	21.05 ± 0.21	24.53 ± 0.21	22.37 ± 0.11	19.96 ± 0.41	17.45 ± 0.13	21.33 ± 0.12
YI	9.88 ± 0.06	16.33 ± 0.05	20.82 ± 0.13	16.77 ± 0.36	18.58 ± 0.07	22.25 ± 0.16	19.94 ± 0.37	24.32 ± 0.09	21.47 ± 0.27	19.46 ± 0.23	16.42 ± 0.17	20.63 ± 0.02
FF	8.65 ± 0.07	16.60 ± 0.25	21.56 ± 0.04	18.16 ± 0.03	19.14 ± 0.15	22.61 ± 0.18	21.03 ± 0.26	24.82 ± 0.16	22.72 ± 0.46	20.80 ± 0.10	17.12 ± 0.05	20.99 ± 0.13
MF	8.63 ± 0.18	16.68 ± 0.04	20.97 ± 0.04	18.94 ± 0.08	18.88 ± 0.29	22.95 ± 0.06	23.15 ± 0.22	27.66 ± 0.07	23.05 ± 0.24	25.86 ± 0.09	15.88 ± 0.06	21.60 ± 0.09
15 DAP	8.53 ± 0.04	16.29 ± 0.01	21.35 ± 0.10	17.08 ± 0.19	22.30 ± 0.17	22.82 ± 0.09	20.18 ± 0.30	23.53 ± 0.12	22.45 ± 0.06	19.21 ± 0.19	17.35 ± 0.15	20.17 ± 0.01
40 DAP	8.81 ± 0.12	15.53 ± 0.12	20.43 ± 0.22	17.65 ± 0.09	23.30 ± 0.20	23.81 ± 0.10	21.71 ± 0.06	24.57 ± 0.11	23.75 ± 0.09	19.44 ± 0.14	17.02 ± 0.07	19.85 ± 0.09
90 DAP	16.23 ± 0.08	18.30 ± 0.22	23.47 ± 0.11	20.88 ± 0.17	30.47 ± 0.01	25.82 ± 0.06	23.55 ± 0.27	27.46 ± 0.09	23.03 ± 0.19	26.33 ± 0.30	17.64 ± 0.04	23.04 ± 0.21
130 DAP	20.37 ± 0.12	19.30 ± 0.22	23.56 ± 0.18	21.97 ± 0.23	28.58 ± 0.10	23.75 ± 0.07	24.24 ± 0.41	27.13 ± 0.29	23.83 ± 0.27	28.96 ± 0.19	18.53 ± 0.02	24.12 ± 0.14
Average C_t	8.84 ± 4.76	16.66 ± 1.25	21.77 ± 1.11	18.55 ± 1.72	19.42 ± 4.72	23.35 ± 0.85	22.51 ± 1.58	25.36 ± 1.41	23.53 ± 1.02	21.13 ± 2.98	17.35 ± 0.99	21.31 ± 1.15

SR, seedling root; SS, seedling stem; SYL, seedling young leaf; SML, seedling mature leaf; AR, adult root; AS, adult stem; AYL, adult young leaf; AML, adult mature leaf; IB, inflorescence bud; YI, young inflorescence; MF, male flower; FF, female flower; 15 DAP, seed of 15 days after pollination; 40 DAP, seed of 40 days after pollination; 90 DAP, seed of 90 days after pollination; 130 DAP, seed of 130 days after pollination. Values are mean ± SD; standard deviation of three independent technical.

Table S2. Stability ranking of candidate reference genes in different tissues and developmental stages.

Experiment Sets	Ranking Order *	RefFinder		ΔCt		BestKeeper		NormFinder		geNorm	
		Genes	Geomean of Ranking Values	Genes	Average of Standard Deviation	Genes	Standard Deviation [±CP]	Genes	Stability Value	Genes	Stability Value
Seedlings	1	*UCE*	1.495	*UCE*	1.223	*PLA*	0.277	*UCE*	0.033	*ACT* | *UCE*	0.067
	2	*ACT*	2.000	*ACT*	1.227	*18S*	0.311	*ACT*	0.034	-	-
	3	*PLA*	3.027	*CYC*	1.272	*TEF2*	0.385	*EF1α*	0.267	*PLA*	0.393
	4	*18S*	4.356	*PLA*	1.291	*ACT*	0.527	*RPS13*	0.571	*18S*	0.488
	5	*CYC*	4.821	*18S*	1.360	*UCE*	0.584	*CYC*	0.592	*TEF2*	0.527
	6	*TEF2*	5.886	*RPS13*	1.363	*CYC*	0.726	*RPII*	0.688	*CYC*	0.58
	7	*RPS13*	6.055	*EF1α*	1.410	*UBL*	0.736	*PLA*	0.704	*RPS13*	0.630
	8	*EF1α*	6.593	*TEF2*	1.436	*RPS13*	0.847	*TUB*	0.857	*UBL*	0.686
	9	*RPII*	8.349	*RPII*	1.526	*RPII*	1.101	*18S*	0.971	*EF1α*	0.781
	10	*UBL*	8.859	*UBL*	1.631	*EF1α*	1.109	*TEF2*	1.131	*RPII*	0.851
	11	*TUB*	10.156	*TUB*	1.820	*TUB*	1.519	*UBL*	1.402	*TUB*	0.975
	12	*GAPDH*	12.000	*GAPDH*	5.811	*GAPDH*	4.472	*GAPDH*	5.786	*GAPDH*	1.781
Adult plants	1	*RPS13*	2.213	*RPS13*	1.626	*UCE*	0.785	*EF1α*	0.260	*CYC* | *RPS13*	0.273
	2	*CYC*	2.515	*CYC*	1.632	*PLA*	0.854	*ACT*	0.377	-	-
	3	*EF1α*	3.000	*EF1α*	1.691	*UBL*	0.899	*RPII*	0.408	*EF1α*	0.599
	4	*UCE*	3.500	*RPII*	1.700	*CYC*	1.024	*RPS13*	0.537	*RPII*	0.647
	5	*RPII*	4.427	*UCE*	1.712	*TEF2*	1.084	*CYC*	0.606	*UCE*	0.699
	6	*ACT*	4.736	*ACT*	1.756	*RPS13*	1.223	*UCE*	0.796	*ACT*	0.763
	7	*PLA*	5.803	*UBL*	1.844	*ACT*	1.346	*PLA*	1.023	*UBL*	0.810
	8	*UBL*	5.856	*TEF2*	1.975	*RPII*	1.470	*UBL*	1.131	*TEF2*	0.861
	9	*TEF2*	7.326	*PLA*	1.977	*EF1α*	1.595	*TEF2*	1.292	*PLA*	0.944
	10	*TUB*	10.000	*TUB*	2.488	*TUB*	2.194	*TUB*	1.675	*TUB*	1.158
	11	*GAPDH*	11.000	*GAPDH*	4.806	*GAPDH*	4.562	*GAPDH*	4.531	*GAPDH*	1.786
	12	*18S*	12.000	*18S*	5.350	*18S*	4.575	*18S*	5.129	*18S*	2.380

Table S2. *Cont.*

Experiment Sets	Ranking Order *	RefFinder		ΔCt		BestKeeper		NormFinder		geNorm	
		Genes	Geomean of Ranking Values	Genes	Average of Standard Deviation	Genes	Standard Deviation [±CP]	Genes	Stability Value	Genes	Stability Value
Flower development	1	*PLA*	1.861	*PLA*	0.836	*ACT*	0.182	*UCE*	0.076	*ACT* | *GAPDH*	0.157
	2	*ACT*	1.968	*UCE*	0.842	*PLA*	0.199	*PLA*	0.133	-	-
	3	*UCE*	2.378	*ACT*	0.880	*GAPDH*	0.231	*TEF2*	0.199	*PLA*	0.220
	4	*GAPDH*	2.913	*GAPDH*	0.900	*UCE*	0.330	*EF1α*	0.397	*UCE*	0.247
	5	*TEF2*	4.401	*TEF2*	0.902	*18S*	0.479	*ACT*	0.467	*TEF2*	0.343
	6	*EF1α*	6.447	*EF1α*	1.041	*TEF2*	0.480	*GAPDH*	0.536	*CYC*	0.418
	7	*CYC*	6.964	*CYC*	1.085	*CYC*	0.487	*RPII*	0.762	*UBL*	0.498
	8	*UBL*	8.426	*RPII*	1.221	*UBL*	0.567	*CYC*	0.875	*EF1α*	0.599
	9	*RPII*	8.651	*UBL*	1.263	*EF1α*	0.762	*RPS13*	1.101	*18S*	0.686
	10	*18S*	8.779	*18S*	1.379	*RPII*	0.930	*UBL*	1.148	*RPII*	0.798
	11	*RPS13*	10.462	*RPS13*	1.431	*RPS13*	1.163	*18S*	1.204	*RPS13*	0.914
	12	*TUB*	12.000	*TUB*	2.639	*TUB*	2.170	*TUB*	2.615	*TUB*	1.202
Seed development	1	*UCE*	2.376	*UCE*	1.586	*UBL*	0.449	*UCE*	0.217	*RPII* | *RPS13*	0.490
	2	*RPS13*	2.546	*RPS13*	1.625	*TEF2*	0.525	*EF1α*	0.229	-	-
	3	*RPII*	3.310	*EF1α*	1.632	*PLA*	0.883	*RPS13*	0.350	EF1α	0.677
	4	*EF1α*	3.568	*ACT*	1.641	*CYC*	1.313	*RPII*	0.525	UCE	0.695
	5	*UBL*	4.304	*RPII*	1.660	*ACT*	1.444	*ACT*	0.587	ACT	0.734
	6	*ACT*	4.729	*CYC*	1.702	*RPII*	1.476	*CYC*	0.844	CYC	0.768
	7	*CYC*	5.422	*UBL*	2.169	*RPS13*	1.620	*UBL*	1.832	UBL	0.958
	8	*PLA*	6.260	*PLA*	2.273	*UCE*	1.786	*PLA*	1.892	PLA	1.106
	9	*TEF2*	6.344	*TEF2*	2.450	*EF1α*	2.029	*GAPDH*	2.065	TEF2	1.217
	10	*GAPDH*	9.740	*GAPDH*	2.686	*GAPDH*	3.360	*TEF2*	2.215	GAPDH	1.518
	11	*TUB*	11.000	*TUB*	3.182	*TUB*	4.156	*TUB*	2.874	TUB	1.860
	12	*18S*	12.000	*18S*	4.003	*18S*	4.813	*18S*	3.901	18S	2.217

Table S2. *Cont.*

Experiment Sets	Ranking Order *	RefFinder		ΔCt		BestKeeper		NormFinder		geNorm	
		Genes	Geomean of Ranking Values	Genes	Average of Standard Deviation	Genes	Standard Deviation [±CP]	Genes	Stability Value	Genes	Stability Value
Entire growth cycle	1	*UCE*	1.861	*UCE*	1.570	*PLA*	0.616	*EF1α*	0.287	*ACT* | *UCE*	0.585
	2	*ACT*	2.213	*ACT*	1.576	*UBL*	0.747	*ACT*	0.340	-	-
	3	*EF1α*	3.224	*EF1α*	1.593	*TEF2*	0.779	*UCE*	0.447	*CYC*	0.622
	4	*CYC*	4.162	*CYC*	1.615	*UCE*	0.915	*RPII*	0.542	*EF1α*	0.743
	5	*PLA*	4.450	*RPII*	1.636	*CYC*	0.942	*CYC*	0.742	*RPII*	0.791
	6	*RPII*	5.318	*RPS13*	1.708	*ACT*	0.994	*RPS13*	0.842	*RPS13*	0.830
	7	*UBL*	5.471	*PLA*	1.817	*RPS13*	1.277	*PLA*	1.085	*UBL*	0.913
	8	*RPS13*	6.236	*UBL*	1.837	*RPII*	1.342	*UBL*	1.238	*PLA*	0.961
	9	*TEF2*	6.839	*TEF2*	1.905	*EF1α*	1.445	*TEF2*	1.347	*TEF2*	0.997
	10	*TUB*	10.000	*TUB*	2.615	*TUB*	2.439	*TUB*	1.979	*TUB*	1.261
	11	*18S*	11.000	*18S*	4.220	*18S*	3.406	*18S*	3.946	*18S*	1.781
	12	*GAPDH*	12.000	*GAPDH*	4.285	*GAPDH*	3.920	*GAPDH*	4.022	*GAPDH*	2.198

* The 1st is the most stable, and the 12th is the least stable.
